# Improved protocols for functional analysis in the pathogenic fungus *Aspergillus flavus*

**DOI:** 10.1186/1471-2180-7-104

**Published:** 2007-11-26

**Authors:** Zhu-Mei He, Michael S Price, Gregory R OBrian, D Ryan Georgianna, Gary A Payne

**Affiliations:** 1 Department of Plant Pathology, North Carolina State University, Raleigh, NC 27695, USA; 2 School of Life Sciences, Sun Yat-Sen University, Guangzhou 510275, P. R. China; 3 Department of Molecular Genetics and Microbiology, Duke University Medical Center, Durham, NC 27710, USA

## Abstract

**Background:**

An available whole genome sequence for *Aspergillus flavus *provides the opportunity to characterize factors involved in pathogenicity and to elucidate the regulatory networks involved in aflatoxin biosynthesis. Functional analysis of genes within the genome is greatly facilitated by the ability to disrupt or mis-express target genes and then evaluate their result on the phenotype of the fungus. Large-scale functional analysis requires an efficient genetic transformation system and the ability to readily select transformants with altered expression, and usually requires generation of double (or multi) gene deletion strains or the use of prototrophic strains. However, dominant selectable markers, an efficient transformation system and an efficient screening system for transformants in *A. flavus *are absent.

**Results:**

The efficiency of the genetic transformation system for *A. flavus *based on uracil auxotrophy was improved. In addition, *A. flavus *was shown to be sensitive to the antibiotic, phleomycin. Transformation of *A. flavus *with the *ble *gene for resistance to phleomycin resulted in stable transformants when selected on 100 μg/ml phleomycin. We also compared the phleomycin system with one based on complementation for uracil auxotrophy which was confirmed by uracil and 5-fluoroorotic acid selection and via transformation with the *pyr4 *gene from *Neurospora crassa *and *pyrG *gene from *A. nidulans *in *A. flavus *NRRL 3357. A transformation protocol using *pyr4 *as a selectable marker resulted in site specific disruption of a target gene. A rapid and convenient colony PCR method for screening genetically altered transformants was also developed in this study.

**Conclusion:**

We employed phleomycin resistance as a new positive selectable marker for genetic transformation of *A. flavus*. The experiments outlined herein constitute the first report of the use of the antibiotic phleomycin for transformation of *A. flavus*. Further, we demonstrated that this transformation protocol could be used for directed gene disruption in *A. flavus*. The significance of this is twofold. First, it allows strains to be transformed without having to generate an auxotrophic mutation, which is time consuming and may result in undesirable mutations. Second, this protocol allows for double gene knockouts when used in conjunction with existing strains with auxotrophic mutations.

To further facilitate functional analysis in this strain we developed a colony PCR-based method that is a rapid and convenient method for screening genetically altered transformants. This work will be of interest to those working on molecular biology of aflatoxin metabolism in *A. flavus*, especially for functional analysis using gene deletion and gene expression.

## Background

*Aspergillus flavus *is a ubiquitous fungus and a plant and animal health concern. It can colonize seeds of maize, peanuts, cotton, and tree nuts during development and contaminate them with the carcinogenic secondary metabolite, aflatoxin (AF), which affects food safety and agricultural trade [1]. *A. flavus *is also the second leading cause of aspergillosis in humans and the leading causative agent of chronic indolent invasive sinonasal infection in immunocompetent patients [2].

Due to the significant health and economic impacts of AF contamination, much attention has been paid to the biochemistry, genetics, and molecular biology of the AF biosynthetic pathway [3]. The genes encoding the biosynthetic pathway are clustered in a 70 kb region of the chromosome [4]. Many factors, such as nutrition, pH, temperature, exogenous stress, and fungal development have been observed to affect AF production [5,6]. However, it still remains unclear how these factors influence AF production and how regulatory elements such as the aflatoxin pathway transcription factor *aflR *are controlled.

An available genome sequence of this fungus holds promise in addressing important questions concerning pathogenicity and the regulatory elements involved in AF biosynthesis. Future studies will require an efficient and reproducible *A. flavus *transformation system. A successful transformation system relies on effective selectable markers, a highly efficient and reproducible transformation procedure, and a rapid and reliable screening procedure for transformants. The most commonly used method to select for genetic transformants in *Aspergillus *species utilizes auxotroph complementation. While such selection can be effective, it requires that the recipient strain be mutated for auxotrophy [7] and it is difficult to generate a double mutant strain for genomic analysis.

Marker genes encoding resistance to antibiotics have been used in genetic engineering and molecular biology research in various organisms [8,9]. Unfortunately, *A. flavus *and many other species of *Aspergillus *are relatively resistant to these antibiotics, rendering them unusable as selectable markers. The antibiotic phleomycin has now been widely applied in transformation of plant and animal cells, and fungi [8,10-13]. However, there is no report, to date, utilizing this antibiotic for *A. flavus *transformation. We described here the use of phleomycin resistance combined with uracil auxotrophy for *A. flavus *transformation. This technology allows the creation of double mutant strains and the transformation of prototrophic strains of *A. flavus*.

Gene disruption and overexpression play central roles in the analysis of gene function related to aflatoxin biosynthesis. Homologous recombination is, in principle, the most efficient method of disrupting or replacing a target gene. However, deletion of many genes is not expected to result in a readily detectable phenotype, and no data on the frequency of gene replacement in most organisms (including *A. flavus*) is available. Establishing a simple and reliable method for screening transformants is important for detecting transformants which have the correct gene deletion/replacement from within a large pool of transformants. PCR (Polymerase Chain Reaction) is among the assays that have been applied to the rapid screening of transgenes. DNA extraction is usually the most time-consuming requirement of this process and this can hinder its effective deployment when either a large number of samples need to be analyzed or when there is only a small amount of material for DNA extraction [14]. DNA extraction protocols for fungi [15,16] are inefficient in releasing the cellular contents from mature hyphae or spores. We report a rapid PCR-based screening method for identifying transformants of *A. flavus*.

## Results

### Sensitivity of *A. flavus *to antibiotics

The resistance of *A. flavus *3357 to kanamycin (Sigma) and hygromycin (Sigma) was tested because they have been used widely in other systems and are relatively inexpensive. Spores of *A. flavus *3357 (10^5 ^spores/plate) were inoculated onto MM plates (Czapek Dox medium) supplemented with kanamycin at 100, 500, and 1000 μg/ml, or hygromycin at 500, 1000, and 1500 μg/ml. No sensitivity to either antibiotic was detected at any of the tested concentrations. Similarly, growth of *A. flavus *spores (10^5 ^or 10^7 ^per plate) was not inhibited on YPD medium supplemented with either 70 or 100 μg/ml of nourseothricin. In contrast, growth of *A. flavus *was inhibited on MM medium amended with 50 μg/ml or 100 μg/ml phleomycin. Medium pH affected the activity of phleomycin. No growth was detected at either concentration of phleomycin when the pH of the medium was 7.5; however, inhibition of growth was inconsistent on medium at pH 6.5.

### Phleomycin as a selection marker in *A. flavus *transformation

To examine the potential for phleomycin as a selectable marker for transformants of *A. flavus*, strain 3357 was transformed with pBC-phleo, a construct containing the *ble *gene for phleomycin resistance controlled by the *A. nidulans gpdA *promoter and the *S. cerevisiae CYC1 *terminator [17]. Transformants were clearly visible on phleomycin-containing MLS medium after 2 days of incubation at 37°C. Selection of stable transformants was enhanced at pH 7.5 as compared to pH 6.5. A few colonies were found on the no-DNA transformation control plates when the protoplasts were plated onto MLS medium containing 100 μg/ml of phleomycin at pH 6.5. In contrast, no growth was observed in the control plates at either 50 or 100 μg/ml of phleomycin when the pH was adjusted to pH 7.5

**Figure 1:**
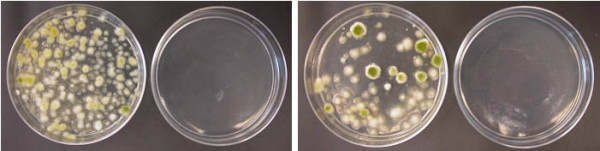
**Transformants of *A. flavus *3357 with pBC-phleo**. Twice as many colonies were observed when transformants were plated on MLS plus 50 μg/ml of phleomycin (left 2 plates) versus 100 μg/ml of phleomycin (right 2 plates). The two plates lacking growth of the fungus are negative controls for untransformed *A. flavus *3357 under the selection of phleomycin at 50 μg/ml (left) or 100 μg/ml (right).

**Figure 2:**
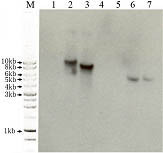
**Southern blot analysis showing the presence of the pBC-phleo vector in two transformants of *Aspergillus flavus***. Lanes 1 and 5: strain 3357-5; lanes 2 and 6: strain 3357-5 transformed with a pBC-phleo alcA::rasA construct: Lanes 3 and 7: strain 3357-5 ΔrdiA complemented with the pBC-phleo vector containing rdiA. Lane 4: Blank. DNA in lanes 1–3 digested with *BamH *I and DNA in lanes 5–7 digested with *Kpn *I. Southern hybridization was performed using a probe amplified from plasmid pBC-phleo with the following primer pairs: BleF, 5'CCCGCTTGAGCAGACATC3' and BleR, 5'TCGTCCTGACTGGCTGCG3'.

The concentration of phleomycin in the regeneration medium also affected the selection of stable transformants. Twice as many colonies were observed when transformants carrying pBC-phleo were plated on MLS plus 50 μg/ml of phleomycin versus 100 μg/ml (Figure 1). To determine the stability of these transformants, 50 colonies from media amended with each phleomycin concentration were plated first on non-selective PDA medium (pH 7.5) and then later transferred to both PDA (pH 7.5) and PDA (pH 7.5) supplemented with 100 μg/ml of phleomycin. Only those transformants that grew equally well on PDA plus phleomycin as on PDA were considered stable transformants. After three transfers, only 1 of 34 colonies from the original plate selected on 50 μg/ml of phleomycin maintained resistance to phleomycin. Conversely, 11 of 18 colonies selected on 100 μg/ml of phleomycin remained phleomycin-resistant after subculturing. Thus both 50 μg/ml phleomycin and 100 μg/ml were inhibitory to fungal growth, but more stable transformants were obtained using 100 μg/ml of phleomycin. When 100 μg/ml of phleomycin was used for selection, we were able to consistently obtain transformants with a single integration of the *ble *gene as shown by Southern analysis (Figure 2).

### Isolation of pyrG mutants of *A. flavus *NRRL 3357

No uracil auxotroph of *A. flavus *strain NRRL 3357 is available. Because the genome sequence is complete for this strain, the selection of a uracil mutant will allow genetic transformation of this strain by established protocols and facilitate studies on its pathogenicity and toxin production. *A. flavus *NRRL 3357 (10^5 ^or 10^7 ^spores) was inoculated onto plates containing MM medium (2% agar) supplemented with 5-fluoroorotic acid (FOA) at 0, 0.5, 1.0, 1.5, or 2.0 mg/ml. No growth was observed on any plates containing FOA. Conidia of *A. flavus *3357 were subjected to NQO mutagenesis and incubated on MMU containing 1.5 or 2.0 mg/ml of FOA at 30°C for one week. A total of 110 colonies were randomly selected from both FOA-containing media. The colonies were cultured on either MMU or MM simultaneously for three generations, and then transferred to four different media (MM, MMU, MM + 2 mg/ml FOA, and MMU + 2 mg/ml FOA) to confirm and identify *pyrG *mutants. Six colonies originally selected on 1.5 mg/ml FOA grew on all four media, indicating they were FOA resistant mutants but not uracil auxotrophs. In contrast eight colonies originally selected on medium contained 2.0 mg/ml FOA grew on MMU and MMU plus FOA but not on MM and MM plus FOA, indicating they were FOA resistant mutants and uracil auxotrophs.

**Figure 3:**
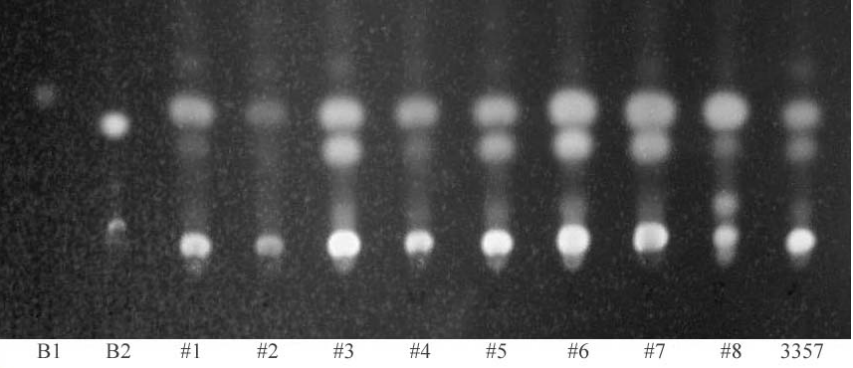
**Visualization of aflatoxin production in strain 3357 and the generated *pyrG *mutants of 3357 by thin layer chromatography (TLC)**. B1: standard for aflatoxin B1 (Sigma). B2: standard for aflatoxin B2 (Sigma). Lanes labeled #1-#8 represent *pyrG *mutants. 3357: original *A. flavus *strain.

Thin layer chromatography (TLC) analysis confirmed that eight colonies selected still possessed the ability to produce aflatoxin (see Figure 3). The ability of the *pyrG *mutant to produce aflatoxin is essential for studies designed to examine aflatoxin biosynthesis. Mutant colonies #3, #5, and #7 were randomly chosen for quantification of AF production using HPLC. These three strains produced aflatoxin concentrations comparable to the original strain 3357 (Table 1). The mutants were designated *A. flavus *strains 3357-3, 3357-5, and 3357-7.

**Table 1:** Aflatoxin analysis by high performance liquid chromatography (HPLC) for selected *A. flavus pyrG *mutants

		Aflatoxin, ng/ml				
		
Strain	pH *	G2	G1	B2	B1	Total
*A. flavus *3357	5.47	0	0	1172.7	23009.0	24182.7
*A. flavus *3357-3	5.30	0	0	1167.9	29618.2	30786.1
*A. flavus *3357-5	5.10	0	0	1109.5	21296.2	22405.7
*A. flavus *3357-7	5.58	0	0	1215.7	34758.7	35974.4

* The pH value of the medium was measured after culturing for 36 h.

**Figure 4:**
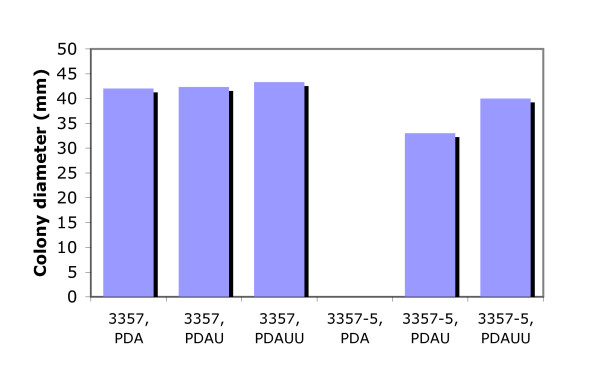
**Growth comparison of *A. flavus *3357 and *pyrG *mutant 3357-5 on either PDA, PDAU or PDAUU**. U: uracil; UU: uracil plus uradine.

Transformation of mutant 3357-5 with the heterologous *pyr4 *gene from *Neurospora crassa *and *pyrG *gene from *A. nidulans *was performed to further confirm this putative *pyrG *mutant. Transformants of either the *pyr4 *gene or *pyrG *gene exhibited a uracil-prototrophic phenotype, which appeared to be stable for more than 10 generations. Comparison of this strain with the original strain 3357 showed that there were no phenotypic differences when cultured at different temperatures (16°C, 28°C, 37°C, or 42°C) or at different pHs (4.0, 6.0, and 8.0). However, the growth of mutant 3357-5 was a little slower than the wild-type 3357 when cultured on PDA supplemented with only uracil. The growth of the mutant is improved when the medium is supplemented with both uracil and uridine (Figure 4).

Strain 3357-5 was also used for transformation using the phleomycin marker (Figure 2). A gene knockout for a rho-dissociation inhibitor was created in 3357-5 by use of the *pyr4 *gene to complement uracil auxotrophy; this strain was then complemented with the native gene using the pBC-phleo vector. In another transformation using the *ble *gene, strain 3357-5 was transformed with a plasmid designed for overexpression of a GTPase while retaining the *pyrG *mutation.

### Factors affecting transformation of *A. flavus*

We evaluated factors responsible for efficient transformation of *A. flavus *through introduction of circular and linear DNA into *A. flavus *strain 3357-5. Cell wall lysis and protoplasting were accomplished using the mixture of enzymes as described in the Methods section. We found that the composition of the digestion buffer influenced the number of transformants obtained from the transformation protocol. A buffer consisting of 7% NaCl, 10 mM NaPO_3_, and 2.5 mg/ml BSA produced protoplasts which gave a transformation efficiency of 200–300 colonies per μg of pBSK-pyr4 DNA. The addition of BSA to the enzyme solution greatly increased transformation efficiency in this system (data not shown).

Using this protocol we found two types of transformants after 2–4 d of incubation on MLS at 37°C. Type 1 colonies (about 10% of the total) grew fast and sporulated normally. The majority of transformants were type 2 colonies, which failed to grow past the initial growth observed after 24 or 36 h of incubation, and almost none sporulated. To determine whether these two types were stable transformants, individual colonies were picked onto PDAU and incubated for 2–3 d at 30°C. The picked transformants were then transferred to both PDAU and PDA medium, grown for an additional 2–3 d, and serially transferred from PDAU to both PDAU and PDA for 4–5 iterations. More than 90% of the type 1 colonies remained prototrophic for uracil after the serial transfers while only 3% of the type 2 colonies were prototrophs. In an attempt to increase the number of stable type 2 colonies, the following regeneration media were examined: 1.2 M sorbitol; 0.6 M sorbitol; 0.4 M (NH_4_)_2_SO_4 _(MLS); 0.4 M (NH_4_)_2_SO_4 _(MLS, pH to 7.5); 0.2 M (NH_4_)_2_SO_4_; 0.6 M KCl; 0.4 M KCl; 1 M sucrose; and 0.6 M sucrose. None of these media improved the stability of the type 2 transformants. Interestingly, we found similar stable transformation frequencies when either the *pyr4 *or the *pyrG *gene was used to complement the uracil mutation; however, no type 2 colonies were observed in the transformation using *pyrG *for complementation.

### Co-transformation of pyr4 and ble genes and comparison of transformation frequency between these two genes

Co-transformation of *A. flavus *strain 3357-5 with 1 μg pBSK-pyr4 and 1 μg pBC-phleo was performed using MLS regeneration medium (pH7.5) supplemented with either 100 μg/ml phleomycin plus uracil, or 100 μg/ml phleomycin only, or neither phleomycin nor uracil. After 3 d of incubation, 75, 9, and 3 colonies were obtained on MLS + phleomycin + uracil (for *ble *selection), MLS (for *pyrG *selection), and MLS + phleomycin (for co-transformation selection) respectively. We observed that the transformation frequency (stable transformants per μg plasmid) differed between pBSK-pyr4 and pBC-phleo. The transformation frequency of pBC-phleo is much higher than pBSK-pyr4 upon co-transformation and when transformed separately.

### Colony PCR analysis to identify a gene knockout strain

**Figure 5:**
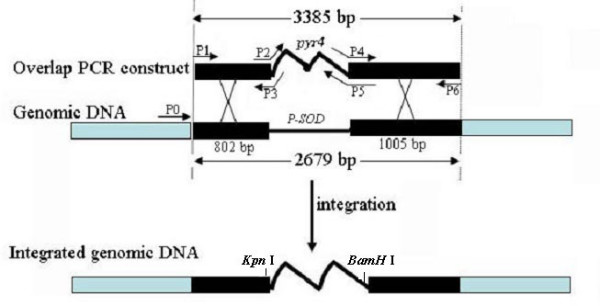
**Overlap PCR construct for transformation of *A. flavus *and the location of primers for identifying transformants using PCR-based screen**. Primers P2, P3, P4, and P5 consist of two parts, one part is homologous to the putative superoxide dismutase (*P-SOD*) gene (GenBank accession no: CA747446) and another part is homologous to selection marker *pyr4*.

**Figure 6:**
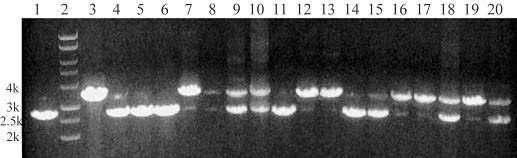
**Colony PCR products for identifying uracil-independent transformants using genomic DNA extracted by CTAB method and primer pairs P1 and P6**. Lane 1: The control native target sequence which PCR-amplified from 3357-5; Lane 3–20: From different transformant colonies.

**Figure 7:**
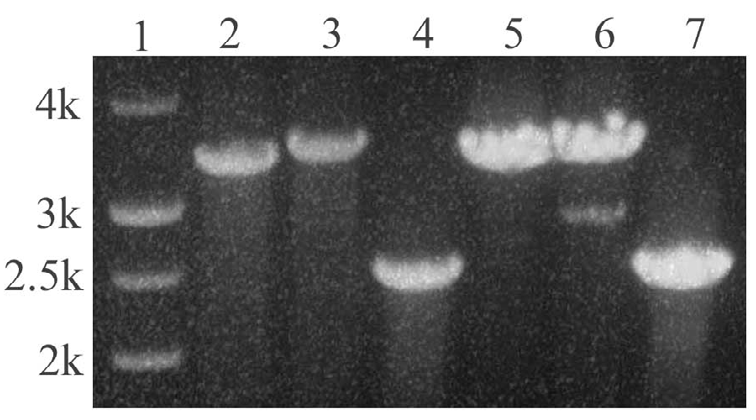
**PCR-amplification of 2 uracil-independent transformants SOD#5 and SOD#30 using different primer pairs to confirm if the transformation construct replaced the target sequence**. Lane 2–4: template DNA from transformant SOD#5 (Lane 3 in Figure 6), Lane 5–7: template DNA from transformant SOD#30 (lane 12 in Figure 6). Primer pairs used as: P1 and P6 were used on lane 2 and 5, band size is 3385 bp; Primers P0 and P6 were used on lane 3 and 6, band size is 3385 + 101 = 3486 bp; Primers P0 and P5 were used on lane 4 and 7, band size is 3385 + 101 -- 1005 = 2481 bp.

**Figure 8:**
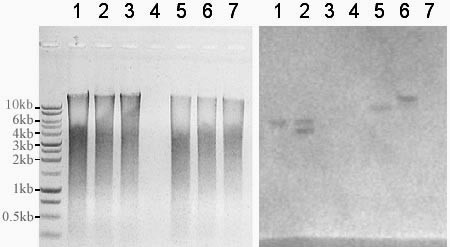
**Southern blot analysis of two *A. flavus *transformants SOD#5 and SOD#30 after deletion of a putative superoxide gene (*GenBank No: ***CA747446**) using an overlap PCR technique with *pyr4 *as a selectable marker**. (A) Genomic DNA digested with *Kpn *I or *BamH *I and run on an 0.8% agrose gel. (B) Southern hybridization using *N. crassa pyr4 *as a probe amplified from plasmid pBSK-pyr4 with the following primer pairs: Pyr-F, 5' TTGGACCACACGAGTCAAG 3' and Pyr4-R, 5' GAAAACGAAATATCCTCCGCC 3'. Lanes 1 & 5 are SOD#5; Lanes 2 & 6 are SOD#30; Lanes 3 & 7 are 3357-5; Lane 4 is blank. Lanes 1–3 digested with *Kpn *I; Lanes 5–7 digested with *BamH *I. The two bands in lane 2 suggest multiple integrations in SOD#30.

A gene predicted to be a superoxide dismutase (GenBank No: CA747446) was deleted in *A. flavus *3357-5 using an overlap PCR technique [18] (Figure 5). Various genomic DNA extraction procedures that included using germinated conidia, heating conidia in a microwave oven, digesting conidia via an enzyme cocktail (lysing enzyme, driselase, β-glucorinidase), treating conidia with SDS plus phenol, or CTAB were attempted. PCR was performed to identify deletion mutants following selection on uracil-deficient medium. We found the CTAB DNA extraction method to be efficient and to yield repeatable results. Figure 6 shows the results of colony PCR from genomic DNA extracted using the CTAB method, using the primer pairs P1 and P6 to identify the transformants. A PCR product of 2679 bp in length identifies the native locus (see lanes 1, 4, 5, 6, 11, and 14 in Figure 6). Alternatively, a PCR product of 3385 bp in length identifies gene replacement by the *pyr4 *marker gene (Figure 6 lanes 3, 12, 13, and 17). The presence of PCR products of 3385 bp and 2679 bp in length indicates that the gene deletion construct inserted ectopically in the *A. flavus *genome (Figure 6 lanes 7, 8, 9, 10, 15, 16, 18, 19, and 20). To further confirm if the target sequence was replaced by *pyr4*, another set of PCR was performed for selected knockout mutants SOD#5 (lane 3 in Figure 6) and SOD#30 (lane 12 in Figure 6) using primer pairs P0 and P5, P0 and P6, and P1 and P6 (Figure 7). We found that the target sequences in these 2 knockout mutants were exactly replaced by the gene deletion construct through homologous recombination. Further Southern blot analysis confirmed that there is only one copy of *pyr4 *in the genome of SOD#5 strain and that strain SOD#30 may have one or more ectopic copies in addition to the site directed integration (Figure 8).

## Discussion

We developed a new transformation protocol for *A. flavus *based on phleomycin resistance. To our knowledge this is the first report of phleomycin being used as a marker for *A. flavus *transformations. One factor important for the success of this procedure is the pH of the regeneration medium containing phleomycin. We found *A. flavus *to be the most sensitive to phleomycin at pH 7.5. Interestingly, this pH differs from the optimum pH for selection of *N. crassa *transformants. Austin *et al*. [10] found pH 5.3–5.8 to give the best selection of *N. crassa *transformants. Also important for the selection of stable transformants using this marker is the concentration of phleomycin in the regeneration medium. While phleomycin at 50 μg/ml resulted in more apparent transformants, they did not maintain stable resistance upon subculturing. We found that regeneration of transformed protoplasts on medium containing 100 μg/ml phleomycin to result in a higher percentage of stable transformants.

Other commonly used chemicals for selection of transformants were examined in our study. Neither hygromycin, kanamycin, nor nourseothricin were found to be toxic to the fungus. Our results on the toxicity of hygromycin differ from those of Ramesh *et al*. who reported the disruption of the serine proteinase gene (*sep*) in *A. flavus *using the hygromycin resistance gene as a selectable marker [19]. This discrepancy may reflect a genetic difference between the two strains examined.

The ability to use phleomycin in a tranformation protocol for NRRL 3357 allows a researcher to avoid using the complementation of an auxotrophic mutation for selection of transformations, which is currently the most commonly used procedure for the *Aspergilli*. This is important for two reasons. First, the generation of auxotrophic mutants often requires a mutagenic agent, which may result in other changes in the strain that may not be readily apparent. Second, auxotrophic strains can be problematic when used in pathogenicity studies. This second point is particularly true if it is desirable to characterize the function of a gene by first doing a gene deletion and then adding a functional gene back in the same strain. Such a comparison would require two auxotrophic mutations, one of which would not be complemented in the gene deletion mutant. The presence of the nutritional mutation may compromise the growth of the gene deletion strain on natural substrates that cannot be supplemented with the necessary nutrient. We demonstrated in this study that phleomycin selection can be used to complement the function of a gene previously deleted in the same strain by a DNA deletion construct containing *pyr4*.

The transformation efficiency differed among the two selectable markers. A percentage of the *pyr4 *transformants formed tiny abortive type 2 colonies, which was reported before in our laboratory [20]. This phenomenon also has been observed in transformation of *A. nidulans *with the heterologous *A. nidulans trpC *gene [21] and *N. crassa pyr4 *gene [22]. In contrast, fewer of the *ble *and the *pyrG *transformants formed type 2 colonies.

In this study also we provide an improved protocol for genetic transformation of *A. flavus*. Regardless of the selective marker, an efficient transformation protocol remains critical to functional analysis. Several conditions were examined in the existing protocol developed in this lab [20]. The most significant increase in transformation resulted from the addition of BSA to the digestive mixture used to release the protoplasts. The addition of BSA in digestion mixture has been used for protoplast isolation of *Tuber borchii*, *Aspergillus *sp., and *Penicillium brevicompactum *[23,24]. The effect of BSA is not always positive, and thus its effect may be species specific. We found the addition of BSA in the protoplast-generating buffer to be critical for increasing the transformation efficiency of *A. flavus*. The positive effect of BSA is unknown, but may be related to the overall quality of the protoplasts from *A. flavus*. The changes made in the protocol consistently improved transformation efficiency of *A. flavus*. Both circular plasmid and linear PCR products were transformed into *A. flavus *with high frequency. We could also easily obtain co-transformants of plasmids containing the phleomycin-resistance marker and plasmids containing the *pyr4 *marker, which provides a powerful tool for the introduction of any non-selectable genetic material into *A. flavus*.

We further tested A. flavus strain 3357-5 to see if it could be used for gene disruption experiments. We generated a pyr4-containing disruption construct of a putative superoxide dismutase gene and used it to transform strain 3357-5. We obtained several transformants and characterized them by PCR. The results from PCR (Figure 6) showed that many of the transformants contained the full length knockout construct. A large percentage of these also contained the native gene indicating ectopic integration. This result is similar to what we have obtained with other knockout constructs. In order to further test for gene disruption, we performed a southern analysis. We chose two transformants, SOD#5 and SOD#30, based on the number of bands generated by PCR. We used a full length pyr4 probe to test for multiple integration events. Consistent with a single site directed integration event, we observed one band with SOD#5. Two bands were observed with SOD#30 indicating one or more ectopic integrations (Figure 8).

Another important consideration for high throughput functional genomics is the selection of transformants because the transformation efficiencies of filamentous fungi remain low compared to yeast [7]. To aid in this process we developed a rapid screening protocol to detect transformants containing transformed DNA. Colony-PCR is a powerful approach because it is less laborious and time-consuming than Southern analysis. For this procedure to be efficient it is necessary to quickly obtain quality template DNA for DNA amplification. Several methods for isolation of DNA from conidia were examined with little success. Fortunately, we were able to obtain high quality DNA from *A. flavus *conidia using the CTAB protocol, which is routinely used to obtain DNA from plants. This is a simple DNA extraction method for PCR-amplification that worked extremely well regardless of the age of the conidia.

The protocols for functional analysis described here make the study of the aflatoxin biosynthetic pathway more straightforward, and may possibly be extended to other filamentous fungi.

## Conclusion

The available genome sequence and whole genome DNA microarrys for *Aspergillus flavus *make it a model system for understanding pathogenicity and mycotoxin production in a seed infecting fungus. To better understand the function of genes in this fungus it is necessary to have an efficient system for gene replacement and complementation. This study reports the use of the antibiotic phleomycin as a dominant selectable marker for transformation and overall improvements in both the transformation protocol and the selection of transformants of *A. flavus*. This transformation protocol provides an efficient and powerful system for functional analysis in this fungus. Previous transformation systems for this fungus rely on auxotrophic markers that require a mutation within the recipient strain. Using the phleomycin transformation protocol no prior mutation is needed. Further, phleomycin can be used in conjunction with other markers to readily disrupt two genes in the same strain. This research will be of interest to those working on pathogenicity and the molecular biology of aflatoxin metabolism in *A. flavus*.

## Methods

### Strains and culture conditions

*Aspergillus flavus *3357 obtained from the Fungal Genetics Stock Center (University of Kansas, Kansas City, USA) and *A. flavus *3357-5 (*pyrG*, tox^+^) were used in this study. The culture media used were PDA (Potato Dextrose Agar, Difco), PDAU (PDA plus 1.12 g/l uracil), PDAUU (PDA plus 1.12 g/l uracil and 1.2 g/l uradine), PDB (Potato Dextrose Broth, Difco), PDBU (PDB plus 1.12 g/l uracil), MM (Czapeck-Dox Broth, Difco), MMU (MM plus 1.12 g/l uracil), YPD (1% yeast extract, 2% peptone, 2% glucose, and 1% agar), MLS (MM plus 0.4 M (NH_4_)_2_SO_4 _and 1% agar). Fungi were grown on solid media at 30°C or shaken in liquid culture at 200 rpm at 30°C when unless otherwise mentioned.

### Making pyrG mutant *A. flavus *3357-5

*A. flavus *strain 3357-5 (*pyrG*, tox^+^) was produced from *A. flavus *3357 by chemical mutation as follows: 0.02 μl NQO (4-nitroquinoline-N-oxide) stock solution (10 mg/ml in acetone) was incubated with conidia (10^6^) in 500 μl of sterile 0.05% Triton X-100 in a microcentrifuge tube and vortexed, followed by incubated at 30°C for 30 min. The reaction was quenched by adding 500 μl of 5% sodium thiosulfate. After vortexing, the mutagenized conidia were incubated at room temperature for 5 min. The conidia were then centrifuged at 1,000 rpm for 3 min, resuspended in 1 ml of 0.05% Triton X-100, and centrifuged again. The final conidial pellet was resuspended in 500 μl of 0.05% Triton X-100. The mutagenized conidia (10^5^) were plated on MMU medium supplemented with 2 g/l of 5-fluoroorotic acid (FOA) and incubated at 30°C. After 5–7 d, FOA resistant colonies were transferred using toothpicks to MM and MMU simultaneously and incubated at 30°C. After 2–3 d, those colonies which grew on MMU but not on MM were transferred again to MM and MMU simultaneously; this selection was repeated 3 times. Finally, the stable FOA-resistant mutants were transferred to MM, MM+FOA, MMU and MMU+FOA to test the ability to grow in the absence of uracil and the sensitivity to FOA.

### Aflatoxin measurement

AF production was observed using thin layer chromatography (TLC) and high performance liquid chromatography (HPLC). 10^8 ^conidia each of *A. flavus *3357 and the subsequent *pyrG *mutants were inoculated in 100 ml PDBU and incubated at 30°C, 200 rpm for 30–36 h. For TLC, AF was extracted and visualized on silica gel plates as described by Hicks *et al*. [25]. HPLC analysis was performed by the Mycotoxin Research Laboratory at NCSU using culture media filtered through Whatman paper by vacuum pump.

### DNA constructs for transformation studies

pBSK-*pyr4 *and pBSK-*pyrG *were constructed by insertion of the fragment carrying the uracil biosynthetic gene *pyr4 *from *N. crassa *or *pyrG *from *A. nidulans *into pBluescript SK^-^. An overlap PCR construct (Figure 5) was constructed according to Davidson *et al*. [18]. All primers used in constructing the overlap PCR construct are listed in Table 2 and shown in Figure 5. This construct contained a *pyr4 *gene, which replaced a putative superoxide dismutase gene (GenBank accession no: CA747446) in *A. flavus *genome, a gene homologous to *sodM *gene in *A. oryzae *(GenBank accession no: AB078724) [26]. Plasmid pBC-phleo, obtained from the Fungal Genetics Stock Center [27], carries the phleomycin resistance cassette in which the *ble *gene is under the control of the *A. nidulans gpdA *promoter and the *Saccharomyces cerevisiae CYC1 *terminator [17]. DNA was isolated using a Wizard Plus SV Minipreps Kit (Promega).

**Table 2:** Primers used in this study

Primer name	Sequence*	Purpose**
P0	TTGAAGTCATGGTGGAGGTG	1
P1	ATGTACTCCGTACTCGGTTG	1, 2
P2	TTATATTCTCCCTCGCCATActtggaccacacgagtcaag	1, 2
P3	cttgactcgtgtggtccaagTATGGCGAGGGAGAATATAA	1, 2
P4	gcggaggatatttcgttttcATAACAAGGCCTCGTATGTG	1, 2
P5	CACATACGAGGCCTTGTTATgaaaacgaaatatcctccgc	1, 2
P6	ACTACACCTTGGGGACTGTA	1, 2

* Sequence in upper-case is homologous to *A. flavus *genome, and lower-case is homologous to *pyr4 *gene of *N. crassa*.

** Purpose 1: identifying transformants; purpose 2: constructing overlap PCR construct.

### Preparation of protoplasts and transformation protocol

Conidia (10^9 ^per flask) were placed into 500-ml flasks containing 100 ml PDBU and incubated at 30°C on a rotary shaker at 200 rpm for 12–16 h. The cultures were then centrifuged at 3,000 rpm for 10 min, washed with sterile water and centrifuged again to completely remove the growth medium. Enzyme solution was added to the fungal tissue (15 ml enzyme solution per 5 g tissue was added and contained 1.2 M NaCl, 10 mM NaPO_3 _(pH 7.0), 70 mg lysing enzyme (Sigma), 40 mg Driselase (Sigma), 0.15 ml β-glucorinidase (98,000 u/ml, Sigma), and 40 mg BSA). This mixture was then shaken at 70 rpm, 30°C for 3 h. Formation of protoplasts was monitored microscopically.

Cell wall debris was removed by centrifugation at 750 rpm for 1 min. The supernatant was brought up to 50 ml with STC buffer (1.2 M sorbitol, 10 mM Tris-HCl (pH 7.5), and 50 mM CaCl_2_). The protoplasts were then centrifuged at 2,500 rpm for 5 min, washed with 50 ml of STC buffer and centrifuged again. 300–500 μl STC buffer was added to resuspend the protoplast pellet by gentle swirling. Protoplasts were then counted using a hemocytometer and diluted to 10^8 ^protoplasts per ml.

100 μl aliquots of the diluted protoplasts were transferred into 1.5 ml tubes and kept on ice. 1 μg of DNA to be transformed was added to each tube and mixed by tapping. One tube was used as a negative control (no DNA). After incubation on ice for 20 min, 1 ml of a 50% PEG solution (50 g PEG (M_r _3,350), 1 ml of 1 M Tris-HCl (pH 8), and 1 ml of 1 M CaCl_2 _in a final volume of 100 ml) was added, mixed by tapping, and incubated at room temperature for 20 min. 100–300 μl of each transformation was plated on the surface of regeneration MLS medium. The plates were incubated at 37°C for 3–5 d.

### Colony PCR analysis

The transformant colonies were transferred to duplicate selection agar plates with toothpicks. Conidia of each fungal colony were obtained from the surface of the agar medium using 50 μl of 0.05% triton X-100 and placed into a microcentrifuge tube containing some 0.5 mm glass beads and 500 μl of CTAB buffer (2% CTAB, 100 mM Tris-HCl (pH 8.0), 20 mM EDTA (pH 8.0), 1.4 M NaCl, 1% polyvinylpyrrolidone-4000, 40 mM β-mercaptoethanol) [28]. Samples were vortexed using a Disruptor Genie for 2 min. The samples were incubated at 65°C for 15 min, then vortexed for 1 min, and incubated at 65°C for another 15 min. 500 μl of chloroform : isoamyl alcohol (24:1) was added and mixed well by shaking tubes. The samples were then centrifuged for 5 min at maximum speed. The aqueous phase was transferred into newly labeled microcentrifuge tubes. 0.08 volumes of 7.5 M NH_4_OAc and 0.54 volumes of isopropanol were added and mixed well. The genomic DNA was pelleted by centrifugation at maximum speed for 5 min. The DNA pellet was resuspended in 20 μl of water.

5 μl of the DNA extracted from transformants or the recipient strain was used as template for PCR-amplification. The PCR primers used are shown in Table 2 and all PCR reactions were in 50 μl. The PCR protocol used is as follows: denaturation at 94°C for 5 min; 30 cycles of 94°C for 10 s, 55°C for 10 s, and 72°C for 1 min per kb of DNA; 72°C for 5 min. Products of the PCR were visualized on agarose gel.

### Southern blot analysis

Genomic DNA was isolated from cultures grown in PDB or PDBU for 3 d using a DNeasy Plant Maxi Kit from Qiagen. DNA was further purified using a Wizard SV genomic DNA purification column. For this procedure, five times the volume of a solution containing 4.5 M guanidine thiocyanate, 0.5 M potassium acetate was added to the genomic DNA. This solution was passed through a SV minicolumn and washed two times with wash buffer. DNA was eluted with water. Approximately 2 μg of genomic DNA was digested with either *Kpn *I or *BamH *I (Promega) for approximately 6 h. Digested samples were run on an 0.8% agarose gel at 90 V for 1 h. The gel was treated and transferred to a Hybond-N^+ ^nylon membrane (Amersham pharmacia biotech) using standard procedures. The membrane was allowed to dry slightly and then auto-cross linked (UV Stratalinker 1800, Stratgene). The probe was prepared from *pyr4 *or *ble *using the DIG High Prime DNA Labeling and Detection Starter Kit II from Roche. Hybridization and washing was performed using standard procedures. Detection was performed using the chemiluminescent reagents supplied with the Kit followed by autoradiography.

## Authors' contributions

ZMH conceived and designed the study, performed the experiments and drafted the manuscript. MSP, GRO, and DRG gave some suggestions, carried out some of the experiments, and amended the manuscript. GAP participated in coordination and funding for the study, critical evaluation, and amended the manuscript. All authors read and approved the final manuscript.
